# Supplementary material for: An open-source framework for large-scale, flexible evaluation of biomedical text mining systems
Source: J Biomed Discov Collab. 2008 Jan 29;3:1. doi: 10.1186/1747-5333-3-1 (PMC2276192; doi:10.1186/1747-5333-3-1)
Supplement: Additional file 1 — Evaluation platform source code. The additional file contains the software evaluation framework discussed in this paper. The most current release can be downloaded from SourceForge.net [1]. See the accompanying README file for instructions on installation and use. Also included in the distribution is a collection of UIMA wrappers for some commonly used BioNLP tools and annotated corpora. [file 1747-5333-3-1-S1.zip › bionlp_uima_repository-1.0.1/docs/api/edu/uchsc/ccp/util/nlp/annotation/comparison/DocumentLevelAnnotationCreator.html]

DocumentLevelAnnotationCreator (Center for Computational Pharmacology Util/UIMA API)


|  |  |  |
| --- | --- | --- |
| **Overview** \| **Package** \| **Class** \| **Tree** \| **Deprecated** \| **Index** \| **Help** | |  |
| **PREV CLASS**   **NEXT CLASS** | **FRAMES**    **NO FRAMES**     **All Classes** |  |
| SUMMARY: NESTED \| FIELD \| CONSTR \| METHOD | DETAIL: FIELD \| CONSTR \| METHOD |  |


---


## edu.uchsc.ccp.util.nlp.annotation.comparison Class DocumentLevelAnnotationCreator

```
java.lang.Object
  edu.uchsc.ccp.util.nlp.annotation.comparison.DocumentLevelAnnotationCreator
```

---

``` public class DocumentLevelAnnotationCreator extends Object ```

This class creates document-level annotations for a given type. The user can optionally require a slot to be present in order for creation to
occur. This is useful for such tasks as creating document-level protein annotations where you want to require the presence of the entrez\_id\_slot so
that gene name normalization results can be computed. By requiring the presence of the entrez\_id\_slot, only proteins that have such a slot will
have a document-level counterpart created. If no such slot presence is requested, then document-level annotations are generated for all annotations
of the specified type.

**Author:**
:   williamb

---

**Constructor Summary**

`DocumentLevelAnnotationCreator(String mentionType)`
  
          This DocumentLevelAnnotationCreator will generate document-level annotations for all annotations of type mentionType.

`DocumentLevelAnnotationCreator(String mentionType,
Set<String> requiredSlots)`
  
          This DocumentLevelAnnotationCreator will generate document-level annotations for only those annotations that have non-empty slots specified by
the requiredSlots Set.


| **Method Summary** | |
| --- | --- |
| `List<TextAnnotation>` | `createDocumentLevelAnnotations(List<TextAnnotation> inputAnnotations)`             Given a list of TextAnnotations, return a list of corresponding document-level annotations |
| `List<TextAnnotation>` | `createDocumentLevelAnnotations(List<TextAnnotation> inputAnnotations, Set<Integer> annotatorsToIgnore)` |

**Methods inherited from class java.lang.Object**

`clone, equals, finalize, getClass, hashCode, notify, notifyAll, toString, wait, wait, wait`

**Constructor Detail**

### DocumentLevelAnnotationCreator

```
public DocumentLevelAnnotationCreator(String mentionType)
```

:   This DocumentLevelAnnotationCreator will generate document-level annotations for all annotations of type mentionType.

    **Parameters:**: `mentionType` -

---


### DocumentLevelAnnotationCreator

```
public DocumentLevelAnnotationCreator(String mentionType,
                                      Set<String> requiredSlots)
```

:   This DocumentLevelAnnotationCreator will generate document-level annotations for only those annotations that have non-empty slots specified by
    the requiredSlots Set.

    **Parameters:**: `mentionType` -: `requiredSlots` -


**Method Detail**

### createDocumentLevelAnnotations

```
public List<TextAnnotation> createDocumentLevelAnnotations(List<TextAnnotation> inputAnnotations)
```

:   Given a list of TextAnnotations, return a list of corresponding document-level annotations

    :   **Parameters:**: `inputAnnotations` - **Returns:**

---


### createDocumentLevelAnnotations

```
public List<TextAnnotation> createDocumentLevelAnnotations(List<TextAnnotation> inputAnnotations,
                                                           Set<Integer> annotatorsToIgnore)
```


---


|  |  |  |
| --- | --- | --- |
| **Overview** \| **Package** \| **Class** \| **Tree** \| **Deprecated** \| **Index** \| **Help** | |  |
| **PREV CLASS**   **NEXT CLASS** | **FRAMES**    **NO FRAMES**     **All Classes** |  |
| SUMMARY: NESTED \| FIELD \| CONSTR \| METHOD | DETAIL: FIELD \| CONSTR \| METHOD |  |


---
